# Supplementary material for: Reviewing progress in public involvement in NIHR research: developing and implementing a new vision for the future
Source: BMJ Open. 2018 Jul 30;8(7):e017124. doi: 10.1136/bmjopen-2017-017124 (PMC6067369; doi:10.1136/bmjopen-2017-017124)
Supplement: Supplementary file 1 [file bmjopen-2017-017124supp001.docx]

**Appendix 1**

**Review Panel Membership**

Simon Denegri, Chair of Review and NIHR National Director for Patients and the Public / Chair, INVOLVE, NIHR

Tina Coldham, Mental Health User Consultant, Trainer & Researcher / Member of INVOLVE

Dr Stuart Eglin, Regional Director, NHS Research and Development North West, Honorary Visiting Professor, Institute of Psychology, Health and Society, University of Liverpool, Associate Member of INVOLVE

Dr Robert Frost, Policy Director, Medical Advocacy and Policy, GSK

Lynn Kerridge, Chief Executive, NIHR Evaluation, Trials and Studies Coordinating Centre (NETSCC)

Rachel Matthews, Theme Lead for Patient and Public Engagement and Involvement

NIHR CLAHRC North West London

Dr Virginia Minogue, Research lead, NHS England

Tara Mistry, NIHR Advisory Board and Member of INVOLVE/NIHR

Prof Sophie Staniszewska, Vice-Chair of the Review, Associate Member of INVOLVE, Senior Research Fellow, Patient and Public Involvement and Patient Experiences, Warwick Medical School, RCN Research Institute, University of Warwick

Dr Claire Stephenson, Research Support Network Manager, Parkinson’s UK

Derek C. Stewart, OBE, Associate Director for PPI, NIHR Clinical Research Network,

Philippa Yeeles, Head of Patient and Public Involvement, NIHR Central Commissioning Facility (CCF)

Sarah Buckland, (Observer), Director, INVOLVE Co-ordinating Centre

Kay Pattison, (Observer), Research Programmes and Contracts Senior Manager

Research and Development, Department of Health

Kathy Mann (secretariat), NIHR Research Programmes Officer, Research and Development, Department of Health.
