# Supplementary material for: Reviewing progress in public involvement in NIHR research: developing and implementing a new vision for the future
Source: BMJ Open. 2018 Jul 30;8(7):e017124. doi: 10.1136/bmjopen-2017-017124 (PMC6067369; doi:10.1136/bmjopen-2017-017124)
Supplement: Supplementary file 2 [file bmjopen-2017-017124supp002.docx]

**Appendix 2 GRIPP2 Short Form**

| **1: Aim Report the aim of PPI in the study** | The aim of the PPI in the study was to broaden the perspective of the Review to ensure it reflected the patient and public perspective. |
| --- | --- |
| **2: Methods: Provide a clear description of the methods used for PPI in the study** | Patents on the Review Panel shaped the design of the review, contributing to the design of data collection method. Patients particularly emphasised the importance of qualitative data collection to capture experiences and perspectives. Patients contributed to the discussions that identified the need for more than one mechanism to gather views and opinions about the progress of public involvement across NIHR since 2006. This enabled the provision of online and postal contributions and included the use of meetings to gather views too. Patients contributed to the discussion of the results, their interpretation and the development of recommendations. |
| **3: Study results—Report the results of PPI in the study, including both positive and negative outcomes** | The PPI contributed to ensuring the transparency of the process as patient panel members agreed on the process and interpretation of the data. The decision to focus on qualitative data collection meant responders were able to provide in depth data that enabled important insights. Patients were involved in the synthesis of data and the identification of key themes and recommendations. Patients were instrumental in championing recommendations to improve diversity and inclusion. |
| **4. Discussion and conclusions—Comment on the extent to which PPI influenced the study overall. Describe positive and negative effects** | The PPI in the Review was important as it placed patient members at the heart of decision-making at key points in the Review. It influenced all the key decisions undertaken and ensured the Review was co-produced by the entire Review panel. In a future review patient members could have a greater role in the way in which diversity and inclusion shape and are considered within policy reviews. |
| **Reflections/critical perspective Comment critically on the study, reflecting on the things that went well and those that did not, so others can learn from this experience** | The PPI input was important in shaping the aim, conduct and outcomes of the Review. Of particular importance was the decision to collect the rich qualitative data which revealed important experiential insights that may not have been captured in desktop evidence gathering conducted in isolation. Future policy reviews need to carefully consider the intersection between the PPI in the process of policy review and the underpinning evidence in framing the policy messages. |

Staniszewska, S, Brett J, Simera I, *et al*. GRIPP2 reporting checklists: tools to improve reporting of patient and public involvement in research. *BMJ* 2017;358:j3453.

Simultaneously published in Research Involvement and Engagement. 3:13 https://doi.org/10.1186/s40900-017-0062-2
