# Supplementary material for: Reviewing progress in public involvement in NIHR research: developing and implementing a new vision for the future
Source: BMJ Open. 2018 Jul 30;8(7):e017124. doi: 10.1136/bmjopen-2017-017124 (PMC6067369; doi:10.1136/bmjopen-2017-017124)
Supplement: Supplementary file 3 [file bmjopen-2017-017124supp003.docx]

**Appendix 3 Vision, mission, strategic goals and principles for 2025**

| **Vision**  A population actively involved in research to improve health and wellbeing for themselves, their family and their communities. |
| --- |

| **Mission**  The public as partners in everything we do to deliver high quality research that improves the health, wellbeing and wealth of the nation. |
| --- |

| **Strategic goals** |
| --- |
| 1. Opportunities to engage and become involved in research are visible and seized by the public. |
| 1. The experience of patients, service users and carers is a fundamental and valued source of knowledge. |
| 1. Public involvement is a required part of high quality research conducted by researchers and their institutions. |
| 1. Public involvement is locally driven and relevant whilst strategically consistent with the NIHR’s goals |
| 1. Evidence of what works is accessible so that others can put it into practice |
| 1. The NIHR has maintained its global presence and influence for working in partnership with the public. |

| **Principles** |
| --- |
| 1. Building on people’s existing capabilities |
| 1. Promoting mutuality and reciprocity |
| 1. Developing peer support networks |
| 1. Breaking down boundaries |
| 1. Facilitating as well as delivering |
| 1. Recognising people and their experiences as assets |

Adapted from Boyle, D, Slay , J and Stephens L. (2010) *Public Services Inside Out. Putting Co-production into Practice.* NESTA, London
