# Supplementary material for: Reviewing progress in public involvement in NIHR research: developing and implementing a new vision for the future
Source: BMJ Open. 2018 Jul 30;8(7):e017124. doi: 10.1136/bmjopen-2017-017124 (PMC6067369; doi:10.1136/bmjopen-2017-017124)
Supplement: Supplementary file 4 [file bmjopen-2017-017124supp004.docx]

Appendix 4 Going the Extra Mile Recommendations

| Communication  and Information | ￭ A consortium including the NIHR, NHS England, Public Health England and public representation should be established on a time-limited basis to consider the needs of patients and the public for information about research. It should have the ability to develop and test different approaches to providing people with information as part of the care pathway and in different service contexts.  ￭ A single access point or ‘portal’ for enabling patients and the public to access information simply and easily about research how they contribute locally and nationally should be co-produced by the NIHR, NHS England, patients and the public and third sector organisations. NHS badging and placement will be an important to public trust.  ￭ The NIHR should run an annual competition to identify best practice and new ideas in using social media and new technology in public involvement, engagement and participation. |
| --- | --- |
| Culture | ￭ The NIHR should commission the development of a set of values, principles and standards for public involvement. These must be co-produced with the public and other partners. They should be framed in such a way, and with a clear set of self-assessment criteria, so that organisations across the NIHR see their adoption as integral to their continuous improvement in public involvement. The achievements of the public, staff and researchers in promoting and advancing public involvement should be celebrated and acknowledged by the NIHR.  ￭ The strategic goals identified in this report should be included in the NIHR overall strategic plan – otherwise known as Vision, Strategy, Actions, Measures (VSAM). These should be the objectives against which public involvement, engagement and participation are planned and reported across the NIHR health research system. |
| Continuous improvement | ￭ We recommend that INVOLVE builds on its forthcoming report on organisational approaches to learning and development by providing leadership and co-ordination including working with workforce development initiatives across the NIHR. It is clear from our inquiry that the public and researchers need to be better supported to do public involvement. All NIHR leaders, funded researchers and staff should receive an induction in public involvement as part of the overall change programme set out in this document. Public involvement leads across the NIHR should also have their own leadership and development programme and opportunities to network and share good practice.  ￭ We recommend that the NIHR measures success along three indices for the foreseeable future:   - Reach: the extent to which people and communities are engaged, participating and involved in NIHR research including the diversity of this population - Relevance: the extent to which public priorities for research are reflected in NIHR funding and activities - Refinement and improvement: how public involvement is adding value to research excellence as funded by the NIHR.   ￭ The results of the 2014 Research Excellence Framework (REF) should be analysed by INVOLVE for key learnings and ways to develop this evidence base |
|  | for REF2020. Above all, public involvement, particularly in relation to the gaining of knowledge, should have an equal importance to wider forms of engagement and science communication, within the REF 2020 definition of societal benefit for panels that have a service remit.  ￭ An independent review should be commissioned by the NIHR in three years’ time to assess the progress made in taking forward the recommendations in this report. |
| Co-production | The public, researchers and health professionals should be empowered and supported better to work together in the future. In respect of the co-production principles that we have been minded to embrace we recommend that the NIHR consider establishing a co-production taskforce to examine how these can be applied in practice. The taskforce should have the ability to undertake rapid testing of these to establish their importance in delivering research excellence. |
| Connectivity | What’s happening at grassroots level must continue to be the driving force in public involvement. Here we wish to see further support given to work that is *locally inspired and driven whilst strategically* consistent with the NIHR overall goals:  ￭ Regional public involvement, engagement and participation ‘citizen’ forums and strategies should be developed in each of the Academic Health Science Networks (AHSN) geographies. We would expect the NIHR’s Collaborations for Leadership in Applied Health Research and Care (CLAHRCs), Research Design Services (RDSs), Local Clinical Research Networks (LCRNs),  Biomedical Research Centres and Units (BRC/Us) to play a key leadership role in the development of these.  ￭ Regionally, locally and institutionally, NIHR infrastructure (CLAHRCs, BRU/, BRCs, LCRNs etc.) Directors and Boards should support and encourage public involvement leads to identify cross-cutting activity in public involvement and develop joint plans and stable resourcing where relevant. ￭ Regional and local partnerships should be identified by the National Director for Patients and the Public in Research to lead on tackling key challenges in the development of public involvement, beginning with diversity and inclusion. ￭ Building partnerships beyond NIHR boundaries – with service partners, third sector and civic organisations - should be seen as a marker of success in this area and measured appropriately.  ￭ Strengthening and improving the support available to researchers locally and regionally through current delivery mechanisms such as the NIHR Research Design Service. |

|  | Appendix 2 - continued  Recommendations |
| --- | --- |
| Coordination | ￭ Leadership and appropriate governance structures will be vital to ensuring that the future development of public involvement in the NIHR has a clear sense of direction and is accountable. The NIHR National Director for Patients and the Public in Research should establish a leadership group consisting of public contributors, senior researchers, public involvement and engagement leads, and a supporting NIHR-wide public involvement forum of public involvement and engagement leads, to provide consistent and coordinated strategic leadership for public involvement, engagement and participation activities across NIHR and identify clear priorities for resourcing.  ￭ All NIHR Coordinating Centres and infrastructure organisations should have a strategy, framework or plan that covers the promotion and advancement of public involvement, participation and engagement in research. Leadership , accountability and funding for this agenda within organisations must be clear and transparent. Progress should be reported annually, made publicly available and an overview included in the NIHRs annual report. |
| Community | A diverse and inclusive public involvement community is essential if research is relevant to population needs and provides better health outcomes for all. We have been struck by the degree to which researchers and public contributors have encountered barriers when trying to work with different communities and populations. This suggests a system-wide issue that needs considered and careful attention. We would recommend that a specific NIHR workstream be developed in this area in the same way that it has developed other work programmes such as ‘Adding Value’ or ‘Pushing the Pace.’ At a bare minimum, a meeting of NIHR senior leaders and colleagues should be convened in the next 12 months to surface the key issues for wider debate. |
